# Evaluation of the Toxicity and Sublethal Effects of Acetamiprid and Dinotefuran on the Predator *Chrysopa pallens* (Rambur) (Neuroptera: Chrysopidae)

**DOI:** 10.3390/toxics10060309

**Published:** 2022-06-08

**Authors:** Yue Su, Xiangliang Ren, Xiaoyan Ma, Dan Wang, Hongyan Hu, Xianpeng Song, Jinjie Cui, Yan Ma, Yongsheng Yao

**Affiliations:** 1 Key Laboratory of Production and Construction Corps of Agricultural Integrated Pest Management in Southern Xinjiang, College of Agriculture, Tarim University, Aral 843300, China; xjsuyue@163.com; 2 State Key Laboratory of Cotton Biology, Institute of Cotton Research, Chinese Academy of Agricultural Sciences, Anyang 455000, China; renxiangliang@caas.cn (X.R.); maxy_caas@126.com (X.M.); nywangdan@sina.com (D.W.); huhongyan1986@163.com (H.H.); sxp15294@126.com (X.S.); aycuijinjie@163.com (J.C.); 3 Zhengzhou Research Base, State Key Laboratory of Cotton Biology, Zhengzhou University, Zhengzhou 450001, China

**Keywords:** ecotoxicity, life table parameters, biological control agents, sublethal effect, neonicotinoids

## Abstract

Neonicotinoid insecticides affect the physiology or behavior of insects, posing risks to non-target organisms. In this study, the effects of sublethal doses of two neonicotinoid insecticides, acetamiprid and dinotefuran, against *Chrysopa pallens* (Rambur) (Neuroptera: Chrysopidae) were determined and compared. The results showed that acetamiprid and dinotefuran at LD_10_ (8.18 ng a.i. per insect and 9.36 ng a.i. per insect, respectively) and LD_30_ (16.84 ng a.i. per insect and 15.01 ng a.i. per insect, respectively) significantly prolonged the larval stages and pupal stages (except acetamiprid LD_10_), compared to control. In addition, acetamiprid and dinotefuran at LD_30_ significantly prolonged the adult preoviposition period (APOP) and total preoviposition period (TPOP). In contrast, the two insecticides at LD_10_ and LD_30_ had no significant effect on the longevity, fecundity, reproductive days, preadult survival rate (%), intrinsic rate of increase (*r*), net reproductive rate (*R_0_*), and finite rate of increase (*λ*). These results provide a theoretical basis for the rational use of these two insecticides and the utilization and protection of *C. pallens*.

## 1. Introduction

Insecticides and beneficial natural enemies are an important part of integrated pest management (IPM). Where the incidence of pests is high, biological control agents may not always control the pests below the economic threshold, and the application of pesticides may be required. When insecticides and beneficial organisms act on similar pests, the effects overlap, and the interaction between the two usually has a negative impact on the beneficial organism [1]. The widespread use of insecticides, however, has affected the richness and types of beneficial organisms in the ecosystem [2]. Therefore, the side effects of pesticides have received worldwide attention.

Neonicotinoids are systemic insecticides that act on the acetylcholine (nACh) receptors of insects and have developed rapidly on the global market [3]. Neonicotinoid insecticides are usually applied using methods such as spraying, irrigation, and seed treatment [4]. The insecticides can have lethal and sublethal impacts on non-target organisms while poisoning target pests, including insect predators and vertebrates [5]. Neonicotinoids are highly effective in controlling crop pests. However, studies have shown that neonicotinoid insecticides have a long half-life in the environment and can be stored in water and soil for a long time without degradation [4]. This poses a threat to arthropods and environmental safety. Moreover, frequent use of neonicotinoid insecticides has led to the development of resistance in target pests and outbreaks of secondary pests [6]. Many studies have found that neonicotinoids have adverse effects on beneficial arthropod organisms [7,8], including *Aphidius colemani* and *Serangium japonicum* [9,10].

In the field, arthropods can be exposed to neonicotinoid insecticides through sprayed droplets, residues, contaminated pollen and nectar, and contact with plant tissues or contaminated prey [11], thereby affecting habit, spawning, and behavior [12]. High doses of imidacloprid have been shown to reduce feeding, mass gain, thorax growth, and mobility in *Nemobius sylvestris* [13]. *Chrysopa pallens* (Rambur) (Neuroptera: Chrysopidae), a beneficial arthropod, can be found in most agricultural regions of the world [14]. Adults and larvae of Chrysopidae (*Chrysoperla genanigra*, *Chrysopa septempunctata*, *C. pallens*, etc.) are predators; adults have high reproductive output and both adults and larvae prey on a variety of agricultural and forestry pests, such as aphids, coccids, mites, thrips, whiteflies, and lepidopteran larvae [15,16,17,18]. Among the Chrysopidae, *C. pallens* is a potential biological control agent in IPM.

The basic requirement of IPM is to manage pests harmful to crops in a sustainable manner. Therefore, it is necessary to evaluate the side effects of pesticides. Exposure or ingestion of systemic pesticides with sublethal concentrations can cause changes in physiological and various biological characteristics, such as survival rate, developmental duration, longevity, and fecundity [7,19,20]. Thiamethoxam has been shown to have negative effects on the development period and fecundity of *Chrysoperla externa* [21], while deltamethrin is highly toxic to *Chrysoperla carnea* larvae and adults [22]. However, there have been no reports on the risks of neonicotinoid insecticides to *C. pallens*.

In order to explore the potential effect of acetamiprid and dinotefuran on *C. pallens*, it is very important to estimate the sublethal doses of acetamiprid and dinotefuran that pose a risk to *C. pallens*. In the present paper, we evaluated the effects of acetamiprid and dinotefuran on *C. pallens* developmental duration, fecundity, lifespan, and population growth parameters under indoor conditions using life table methods.

## 2. Materials and Methods

### 2.1. Insects and Plant Material

*C. pallens* adults were purchased from Beijing Kuoye Tianyuan Biotechnology Co., Ltd. (Beijing, China). *Megoura crassicauda* (Hemiptera: Aphididae) were collected from the leaves and stems of broad bean plants at the Institute of Cotton Research of the Chinese Academy of Agricultural Sciences (Anyang, China). *M. crassicauda* were maintained on broad beans, while *C. pallens* adults were reared in a cubic insect cage (33 cm length × 33 cm width × 33 cm height). Fresh broad bean plants, growing various instars of *M. crassicauda*, were replaced in the cage every day. Eggs laid by adults of *C. pallens* were transferred to a fresh insect box. Then the eggs hatched and enough aphids were supplied every 24 h until they reached the age required for the experiment. All the insects were kept in a controlled-environment chamber at 25 ± 1 °C, 68 ± 5% RH, and L16:D8 photoperiod.

### 2.2. Insecticides

Acetamiprid (97% TC) and dinotefuran (97% TC) were provided by Anyang Quanfeng Biotechnology Co., Ltd. (Anyang, China).

### 2.3. Toxicity Bioassay

Toxicity bioassays were performed through topical application of acetamiprid and dinotefuran. Each insecticide was diluted in acetone in 7 gradient concentrations (viz. 100.0, 50.0, 25.0, 12.5, 6.3, 3.1, 0 ng a.i. per insect) to evaluate their toxic effects against the 2nd instar larvae of *C. pallens* (<24 h) and determine the sublethal doses. A large number of 2nd instar larvae (<24 h) of similar size were collected for toxicity determination. The larvae were placed in Petri dishes (9 cm diameter, 2 cm height) on ice for 30 s to anesthetize them. A topical drop of 0.5 μL insecticide was applied to the abdomen of each larva using an Arnold automatic micro-applicator (Burkard Manufacturing Co., Ltd., Hertfordshire, UK). The larvae of the control group were treated with acetone only. The treated larvae were cultured in an incubator at 25 ± 1 °C, 68 ± 5% RH, and L16:D8 photoperiod and fed enough live aphids. Mortality data of *C. pallens* were recorded two days after treatment. Each dose was replicated three times using 20 larvae per experiment. Treated larvae were considered dead when they were unresponsive to touch by a brush.

### 2.4. Evaluation of Sublethal Effects of the Insecticides on Second Instar Larvae

A total of 190 eggs (<24 h old) were collected; each egg was Placed in a petri dish and incubated at 25 ± 1 ℃, 68 ± 5% RH, and L16:D8 photoperiod. After the eggs hatched and the larvae grew to the 2nd instar, the larvae were randomly divided into the following groups: four treatment groups (i.e., acetamiprid LD_10_ 8.18 and LD_30_ 16.84 ng a.i. per insect and dinotefuran LD_10_ 9.36 and LD_30_ 15.01 ng a.i. per insect) and one control group (treated with acetone). The sublethal doses of acetamiprid and dinotefuran (LD_10_ and LD_30_) were calculated based on a previous toxicity bioassay. The 2nd instar larvae were treated with insecticides using the protocol described in the preceding section. Acetone-treated larvae were used for control. For the experiment, we used 50, 60, and 80 2nd instar larvae (<24 h old) for control, LD_10_, and LD_30_, respectively. The life table data for acetamiprid and dinotefuran were recorded after 48 h. Each larva was considered a separate replicate, placed in a Petri dish separately, and fed on live aphids on fresh leaves every 24 h. The mortality data and developmental stages of each larva were recorded daily. After the emergence of the adults, the male and female were paired individually and transferred into plastic cups (6.5 cm width, 7.5 cm height) and observed daily to record mortality, adult longevity, preoviposition period, and oviposition. The treated *C. pallens* were fed enough live aphids. All the treated *C. pallens* were cultured in an incubator at 25 ± °C, 68 ± 5% RH, and L16:D8 photoperiod.

### 2.5. Data Analysis

SPSS v.19.0 (SPSS Inc., Chicago, IL, USA) was used to calculate the lethal and sublethal doses of acetamiprid and dinotefuran using data obtained from the toxicity bioassay of *C. pallens* 2nd instar larvae. The effects of sublethal doses of acetamiprid and dinotefuran on the 2nd instar larvae of *C. pallens* were analyzed using the age-stage two-sex life table theory [23,24]. The TWOSEX-MSChart software (http://140.120.197.173/Ecology/, accessed on 5 March 2021) [25] was used to analyze the development of different stages, adult longevity, adult preoviposition period (APOP), total preoviposition period (TPOP), and fecundity. Basic life table parameters were also calculated, including age-specific survival rate (*l_x_*), age-stage-specific survival rate (*S_xj_*), age-stage-specific fecundity (*f_xj_*), age-specific fecundity (*m_x_*), age-specific maternity (*l_x_m_x_*), age-stage-specific reproductivity (*v_xj_*), and life expectancy (*e_xj_*), as were demographic parameters, namely the net reproductive rate (*R_0_*), intrinsic rate of increase (*r*), mean generation time (*T*), and finite rate of increase (*λ*). The standard error (SE) and average value were calculated through 100,000 bootstrap iterations to obtain stable SE estimates [26]. Paired bootstrap test was used to compare all treatments. Bootstrap and paired bootstrap tests were carried out using TWOSEX-MSChart [25]. The SigmaPlot 14.0 (Systat Software Inc., San Jose, CA, USA) software was used to create curves for all population parameters, including survival rate, fecundity, reproductive values, and life expectancy.

## 3. Results

### 3.1. Acetamiprid and Dinotefuran Toxicity on Second Instar Larvae of C. pallens

The toxicity of acetamiprid and dinotefuran on the second instar larvae of *C. pallens* is shown in Table 1. The results showed that the LD_10_, LD_30_, and LD_50_ of acetamiprid against *C*. *pallens* were 8.18, 16.84, and 26.50 ng a.i. per insect, respectively. The corresponding values for dinotefuran were 9.36, 15.01, and 20.27 ng a.i. per insect, respectively. There was no mortality in the untreated control group.

### 3.2. Sublethal Effects of Acetamiprid and Dinotefuran on C. pallens

#### 3.2.1. Effects on the Developmental Period, Longevity, and Reproduction of *C. pallens*

The effects of sublethal doses of acetamiprid and dinotefuran on the developmental period, male and female longevity, and reproduction are shown in Table 2. Acetamiprid at LD_10_ and LD_30_ resulted in the development periods of 2.50 days and 2.25 days, respectively, the corresponding values for dinotefuran at LD_10_ and LD_30_ were 2.43 and 2.48 days, respectively. The results indicated that acetamiprid and dinotefuran significantly prolonged the development time of second instar larvae, compared with the control treatment (2.10 days). Acetamiprid at LD_10_ and LD_30_ and dinotefuran at LD_30_ also significantly extended the development of the third instar larvae to 4.00, 3.98, and 3.86 days, respectively, versus the control at 3.59 days. The time it took to transition into the pupal stage was significantly different among the groups (acetamiprid LD_10_ 11.73 days, dinotefuran LD_10_ 12.68 days, and control 12.30 days). Similarly, the time taken to transition into the preadult stage was significantly higher in the acetamiprid (LD_30_ 24.67 days) and dinotefuran (LD_30_ 24.84 days) treatment groups compared with the control (24.04 days). Exposure to acetamiprid and dinotefuran did not negatively affect the average longevities of females and males.

Acetamiprid at LD_10_ and dinotefuran at LD_30_ significantly extended the APOPs to 8.64 and 8.69 days, respectively, compared with the control (6.78 days). Acetamiprid at LD_10_ and LD_30_ markedly increased the TPOPs to 33.00 and 33.35 days, respectively, while dinotefuran at LD_10_ and LD_30_ markedly increased the TPOPs to 32.83 and 33.69 days, respectively, compared to the control group (30.93 days). There were no significant differences in fecundity and reproductive days with different treatments.

#### 3.2.2. Effect of Acetamiprid and Dinotefuran on the Population Growth Parameters of *C. pallens*

The population growth parameters of *C*. *pallens* after exposure to sublethal doses of acetamiprid and dinotefuran are shown in Table 3. The results showed that the preadult survival rate, intrinsic rate of increase (*r*), net reproductive rate (*R_0_*), mean generation time (*T*), finite rate of increase (*λ*), and gross reproduction rate (GRR) were not significantly different among different groups.

### 3.3. Effects of Acetamiprid and Dinotefuran on C. pallens Demographic Parameters

**Figure 1:**
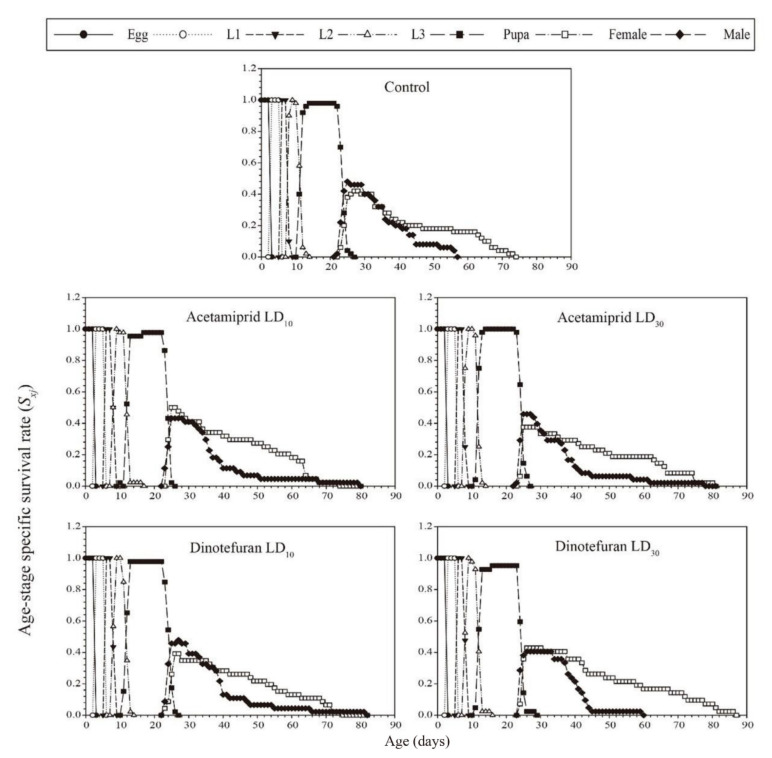
Age-stage-specific survival rate (*Sxj*) of *C. pallens* for 2nd instar *C. pallens* larvae exposed to sublethal acetamiprid and dinotefuran doses.

The age-stage-specific survival rate (*S_xj_*) curve is shown in Figure 1. The results showed that the peak survival rates of females and males in the control group were 42% and 48%, respectively. The peak survival rates for the acetamiprid (i.e., LD_10_: 50% for females and 43.2% for males; LD_30_: 37.5% for females and 45.8% for males) and dinotefuran (i.e., LD_10_: 39.1% for females and 45.7% for males; LD_30_: 42.9% for females and 40.5% for males) treatment groups were similar to the control.

**Figure 2:**
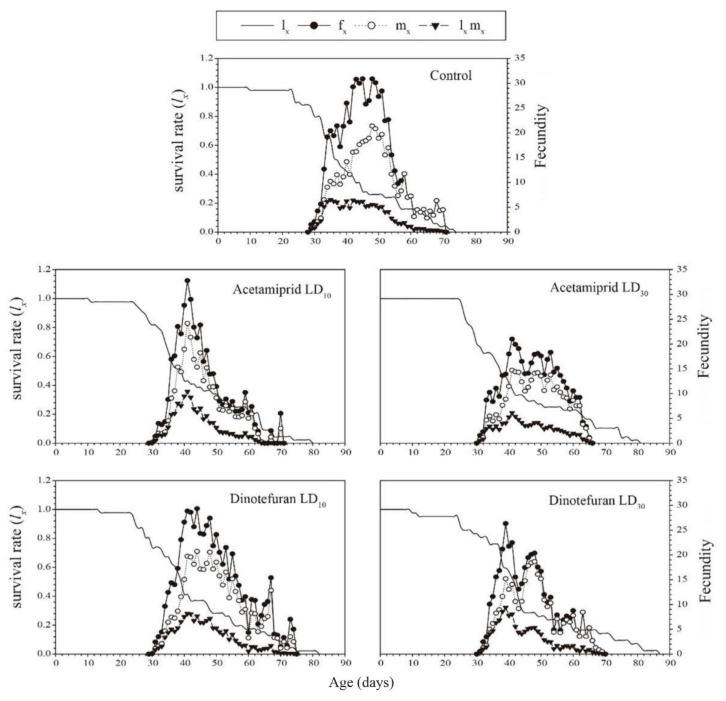
Age-specific survival rate (*l_x_*), female age-specific fecundity (*f_x_*), age-specific fecundity (*m_x_*), and age-specific maternity (*l_x_m_x_*) for 2nd instar *C. pallens* larvae exposed to sublethal acetamiprid and dinotefuran doses.

Graphs for *l_x_* (age-specific survival rate), *f_xj_* (age-stage-specific fecundity), *m_x_* (age-specific fecundity), and *l_x_ m_x_* (net maternity) are shown in Figure 2. On the 30th day, *l_x_* of the control group (0.80) was higher than that of the acetamiprid LD_30_ (0.68) and dinotefuran LD_10_ (0.74) treatment groups. At the age of 47 days, the highest calculated value of *f_xj_* in the control group was 30.88 eggs female^−1^ day^−1^. In acetamiprid treatment, the highest calculated values of *f_xj_* were 32.79 eggs female^−1^ day^−1^ at the age of 40 days and 19.92 eggs female^−1^ day^−1^ at the age of 41 days for LD_10_ and LD_30_, respectively. In dinotefuran treatment, the highest calculated values of *f_xj_* were 28.85 eggs female^−1^ day^−1^ at the age of 40 days and 26.33 eggs female^−1^ day^−1^ at the age of 38 days for LD_10_ and LD_30_, respectively. The peak *m_x_* value appeared at the age of 48 days (20.84 eggs individual^−1^ day^−1^) in the control group. However, the peak *m_x_* values for acetamiprid occurred at 40 and 41 days with 24.16 and 14.39 eggs individual^−1^ day^−1^ for LD_10_ and LD_30_, respectively) for LD_10_ and LD_30_, respectively. The peak *m_x_* values for dinotefuran occurred at 41 and 48 days with 19.74 and 18.67 eggs individual^−1^ day^−1^ for LD_10_ and LD_30_, respectively. However, the net maternity (*l_x_m_x_*) curves of the control group and the treatment groups were not significantly different.

**Figure 3:**
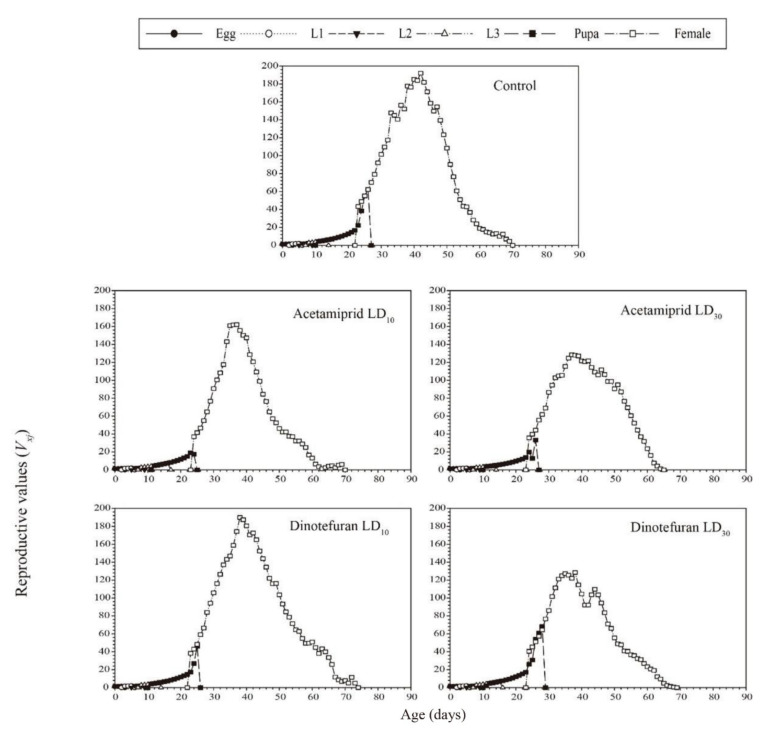
Age-stage-specific reproductive values (*V_xj_*) values of 2nd instar *C. pallens* larvae exposed to sublethal acetamiprid and dinotefuran doses.

The age-stage-specific reproductive value (*v_xj_*) curves are the contribution of each individual to the future reproduction of the entire population at stage *j* of age *x*. *v_xj_* curves of two pesticides at sublethal doses are shown in Figure 3. These results indicated that the effects of acetamiprid and dinotefuran on *C. pallens* reproductive value increased significantly with an increase in dosage. The highest peak for the controls occurred on the 42nd day (191.93 day^−1^). However, the highest peaks in the treatments of acetamiprid at LD_10_ and LD_30_ occurred on the 37th day (162.06 day^−1^) and 37th day (128.46 day^−1^), respectively. The corresponding values for dinotefuran treatments at LD_10_ and LD_30_ were 189.84 day^−1^ (occurred on the 38th day) and 127.22 day^−1^ (occurred on the 35th day), respectively.

**Figure 4:**
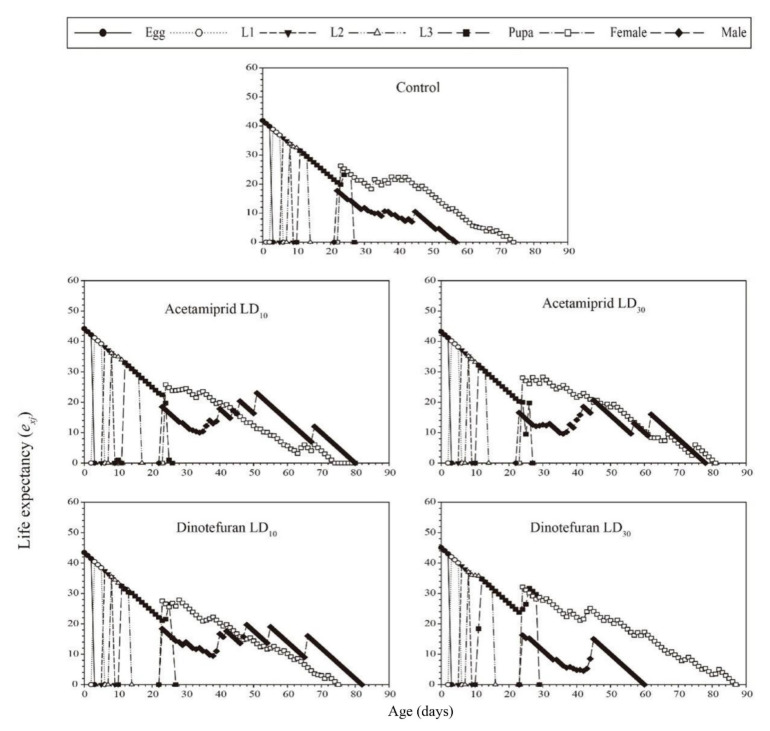
Life expectancy (*e_xj_*) values for 2nd instar *C. pallens* larvae exposed to sublethal acetamiprid and dinotefuran doses.

The age-stage-specific life expectancy (*e_xj_*) is defined as the number of days that individuals of age *x* and stage *j* can continue to live. The results of *e_xj_* are shown in Figure 4. The *e_xj_* values of *C. pallens* treated with acetamiprid at LD_10_ and LD_30_ were 42.20 and 43.19 days, respectively. The corresponding values for dinotefuran treatment at LD_10_ and LD_30_ were 43.46 and 45.05, respectively, while the value for the control group was 41.88 days.

## 4. Discussion

Neonicotinoid insecticides are used extensively in agriculture to control insect pests and also indirectly affect non-target organisms [27,28]. It is reported that *Bemisia tabaci*, *Aphis gossypi*, *Nilaparvata lugens*, and *Myzus persicae*, have developed resistance to neonicotinoid insecticides, to a level that impairs the efficacy of these insecticides [29,30]. The agricultural use of various neonicotinoid insecticides to control piercing pests indirectly threatens the safety of non-target organisms [31,32]. For example, thiamethoxam-treated seeds reduced the fertility of eggs in the F0 and F1 generations and prolonged the pupal stage of the F1 generation of *C. externa* [21]; chlorantraniliprole, cyantraniliprole, and spinetoram treatments decreased the larvae and adult survival rate of *C. carnea* and *Chrysoperla johnsoni* [33]. Therefore, it is very important to evaluate the side effects of pesticides on natural enemies.

In this research, we demonstrated that acetamiprid and dinotefuran were toxic against *C. pallens* and that sublethal doses of acetamiprid and dinotefuran prolonged the larval and preadult stages. These findings suggested that neonicotinoid insecticides such as acetamiprid and dinotefuran have negative effects on the growth and development of *C. pallens*. Similarly, exposure of *Coccinella septempunctata* L. to glass tubes coated with a clothianidin solution extended the developmental time of second instar larvae, third instar larvae, and pupal stage [34]. Dipping of *C. externa* in acetamiprid had negative effects on eggs and first instar larvae [31], while acetamiprid treatment significantly increased the larval stage of *Amblyseius cucumeris* [35].

Some insecticides affect insect behavior, including probing, feeding, and oviposition [12], and prolong growth and development [36,37]. In our study, we found that exposure to acetamiprid and dinotefuran significantly prolonged the APOP and TPOP of *C. pallens*, which was consistent with a report stating that thiamethoxam treatment negatively affected the APOP and TPOP of *Hippodamia variegata* [38]. Further studies are required to determine the mechanisms underlying the effects of acetamiprid and dinotefuran against *C. pallens*.

Acetamiprid and dinotefuran had no significant negative effects on adult longevity, fecundity, oviposition days, preadult survival rate, *r*, *λ*, *R_0_*, *T*, and GRR of the *C. pallens* population. Our results are consistent with the results of other studies: imidacloprid had no negative effects on the fertility, *R_0_, r,* and *T* of *Ceraeochrysa cubana* [39]; imidacloprid and thiamethoxam had no significant effects on the population parameters (*R_0_*, *r*, and *T*) of *Iphiseiodes zuluagai* [40]; and thiamethoxam had no significant effect on adult longevity and fecundity of *H. variegata* [38]. However, other studies have reported that neonicotinoid insecticides induce great damage to beneficial arthropods. Studies revealed that imidacloprid reduced the population growth parameters (*R_0_*, *r*, and *λ*) of *Ceratomegilla undecimnotata* and *H. variegate* [41,42]. These findings indicate that different insecticides can induce different side effects against different insects. Therefore, it is difficult to assess the impact of pesticides on natural enemy insect populations.

Acetamiprid and dinotefuran have less negative impacts on the two-sex life table parameters of *C. pallens*, reflecting their lack of adverse effects on population growth. However, neonicotinoid treatment increased the life expectancy (*e_xj_*) of *C. pallens* in our study, which was not consistent with the report that sublethal concentrations of imidacloprid decreased the adult longevity of *H. variegata* [42]. These differences may be due to different modes of action applied by the different insecticides resulting in different effects on population parameters.

In summary, acetamiprid and dinotefuran have potential adverse effects on *C. pallens*, including negative effects on developmental stage, APOP, and TPOP, but no adverse effects on some life table parameters (*r*, *R_0_*, *λ*, *T*). Although acetamiprid and dinotefuran have less effect on *C. pallens*, insecticide applications should be performed carefully to minimize impacts on non-target organisms. Therefore, it is necessary to further evaluate the toxicity of these two insecticides to *C. pallens* under field or semi-field conditions.

## Figures and Tables

**Table 1:** Toxicity of acetamiprid and dinotefuran on the second instar larvae of *C. pallens*.

Insecticide	N ^a^	Dose (ng a.i. per Insect) (95% CL) ^b^			Slope ± SE ^c^ Data	χ2 ^d^
		LD_10_	LD_30_	LD_50_		
Acetamiprid	420	8.18 (5.74~10.44)	16.84 (13.79~19.67)	26.50 (22.95~30.48)	4.30 ± 0.45	1.65
Dinotefuran	420	9.36 (7.55~10.96)	15.01 (13.18~16.77)	20.27 (18.29~22.36)	6.55 ± 0.61	6.18

^a^ Insect number; ^b^ 95% confidence limits; ^c^ standard error; ^d^ chi-square value (χ^2^).

**Table 2:** Sublethal effects of acetamiprid and dinotefuran on development period, adult longevity, and reproduction of *C. pallens* adults exposed to insecticide from the 2nd instar larval stage.

Stage or Development Period	Control	Acetamiprid LD_10_	Acetamiprid LD_30_	Dinotefuran LD_10_	Dinotefuran LD_30_
Developmental period (days)					
Second instar	2.10 ± 0.04 c	2.50 ± 0.51 a	2.25 ± 0.44 b	2.43 ± 0.50 ab	2.48 ± 0.51 a
Third instar	3.59 ± 0.08 b	4.00 ± 0.81 a	3.98 ± 0.568 a	3.78 ± 0.59 ab	3.86 ± 1.12 a
Pupa	12.30 ± 0.11 b	11.73 ± 0.12 c	12.55 ± 0.01 ab	12.68 ± 0.13 a	12.32 ± 0.01 b
Preadult	24.04 ± 0.14 b	24.29 ± 0.10 b	24.67 ± 0.12 a	24.61 ± 0.16 ab	24.84 ± 0.16 a
Adult longevity (days)					
Female	49.29 ± 3.45 a	49.77 ± 3.25 a	52.36 ± 4.04 a	51.37 ± 3.69 a	56.21 ± 4.23 a
Male	39.70 ± 1.80 a	41.47 ± 2.89 a	39.53 ± 2.56 a	41.37 ± 2.65 a	40.38 ± 1.57 a
Reproduction					
APOP (days)	6.78 ± 0.45 b	8.64 ± 0.76 a	8.28 ± 0.81 ab	7.91 ± 0.59 ab	8.69 ± 0.66 a
TPOP (days)	30.93 ± 0.50 b	33.00 ± 0.79 a	33.35 ± 0.94 a	32.83 ± 0.72 a	33.69 ± 0.65 a
Fecundity (eggs/female)	328.33 ± 87.94 a	255.92 ± 64.41 a	248.72 ± 67.49 a	355.08 ± 109.69 a	273.87 ± 79.81 a
Reproductive days (days)	18.07 ± 3.35 a	17.57 ± 2.14 a	16.51 ± 2.90 a	20.67 ± 3.32 a	16.54 ± 2.72 a

Data are expressed as the mean values ± SE (standard error), calculated by the bootstrap technique with 100,000 resamplings. The means of development period, adult longevity, and reproduction of *C. pallens* followed by different letters in the same row are significantly different among different treatments by the paired bootstrap test based on the confidence interval of difference (*p* < 0.05).

**Table 3:** Sublethal effects of acetamiprid and dinotefuran on the population parameters (mean ± SE) of *C. pallens* adults exposed to insecticide from the 2nd instar larval stage.

Population Parameters	Control	Acetamiprid LD_10_	Acetamiprid LD_30_	Dinotefuran LD_10_	Dinotefuran LD_30_
Preadult survival rate (%)	90.01 ± 4.25 a	93.18 ± 3.78 a	87.48 ± 0.05 a	89.14 ± 4.60 a	88.10 ± 4.99 a
*r*, Intrinsic rate of increase (day^−1^)	0.12 ± 0.01 a	0.12 ± 0.01 a	0.11 ± 0.01 a	0.11 ± 0.01 a	0.12 ± 0.01 a
*R_0_*, Net reproductive rate (offspring per individual)	137.80 ± 42.93 a	127.95 ± 37.24 a	103.72 ± 32.78 a	146.73 ± 51.57 a	123.96 ± 41.38 a
*T*, Mean generation time (days)	40.69 ± 0.84 a	41.55 ± 0.72 a	42.72 ± 1.06 a	42.87 ± 1.13 a	41.33 ± 0.78 a
*λ*, Finite rate of increase (day^−1^)	1.13 ± 0.01 a	1.12 ± 0.01 a	1.11 ± 0.01 a	1.12 ± 0.01 a	1.12 ± 0.01 a
GRR, Gross reproduction rate (offspring/individual)	422.18 ± 104.05 a	314.43 ± 76.87 a	314.04 ± 84.89 a	440.44 ± 133.53 a	321.88 ± 99.16 a

Data are expressed as the mean values ± SE (standard error), calculated by the bootstrap technique with 100,000 resamplings. The means of population parameters of *C. pallens* followed by same letters in the same row are not significantly different among different treatments by the paired bootstrap test based on the confidence interval of difference (*p* < 0.05).

## Data Availability

The data presented in this study are available in article.
